# Supplementary material for: Raptors avoid the confusion effect by targeting fixed points in dense aerial prey aggregations
Source: Nat Commun. 2022 Aug 23;13:4778. doi: 10.1038/s41467-022-32354-5 (PMC9399121; doi:10.1038/s41467-022-32354-5)
Supplement: Supplementary file 1 — Supplementary Information [file 41467_2022_32354_MOESM1_ESM.pdf]

Supplementary Information accompanying:

# Raptors avoid the confusion effect by targeting fixed points in dense aerial prey aggregations

Caroline H. Brighton[†1], Laura N. Kloepper[2,3], Christian D. Harding[1,4], Lucy Larkman[1], Kathryn McGowan[2], Lillias Zusi[2], Graham K. Taylor[†1]

[1]Department of Biology, University of Oxford, 11a Mansfield Road, Oxford, OX1 3SZ, UK
[2]Department of Biological Sciences and Center for Acoustics Research and Education, Spaulding Hall, University of New Hampshire, Durham, NH 03824, USA
[3]Department of Biology, Saint Mary's College, 262 Science Hall, Notre Dame, IN 46556, USA
[4]Department of Physiology, Anatomy, and Genetics, University of Oxford, Sherrington Building, Parks Road, Oxford, OX1 3PT, UK

[†]Corresponding authors: caroline.brighton@biology.ox.ac.uk, graham.taylor@biology.ox.ac.uk

This PDF contains:

Figures S1-S5
Tables S1-S3
References

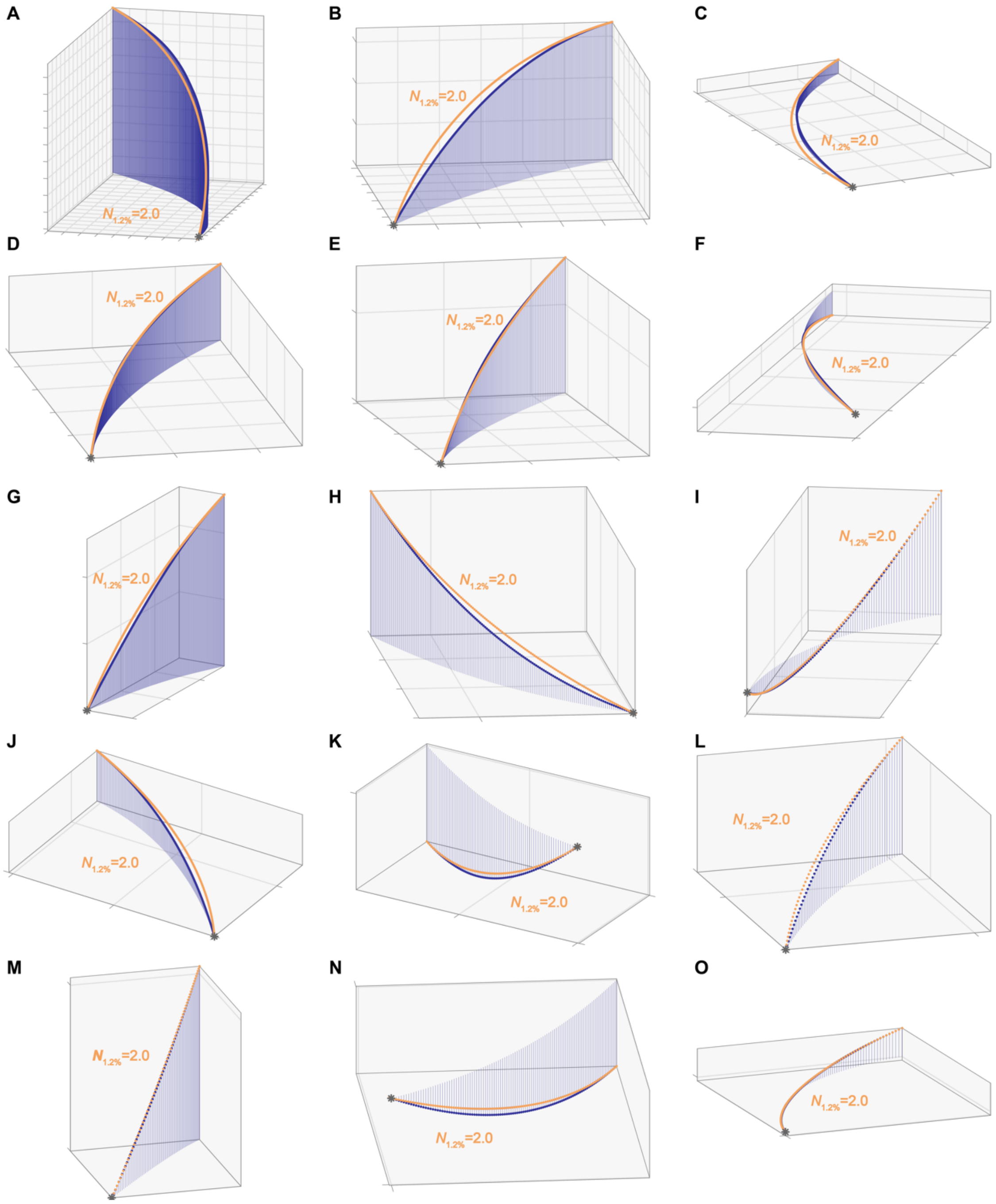


**Figure S1. Long-range approaches of Swainson's Hawks attacking Mexican Free-tailed Bats modelled as constant radius turns into the swarm. (A-O)** Each panel plots the reconstructed three-dimensional attack trajectory of an incoming hawk (dark blue points); dark blue lines are dropped vertically from each point to accurately convey the three-dimensional shape of the trajectory; grey starburst shows the point of capture or near-miss. It was not possible to track the bats that the hawks grabbed at this range, but orange lines plot simulations of the hawk's flight trajectory generated under delay-free PN guidance at a fixed navigation constant of $N = 2$, assuming flight at the same speed as measured and treating the hawk's final position as the target. This serves to generate a constant radius turn that satisfies the kinematic constraint of passing through the hawk's initial and final positions whilst also matching its initial flight velocity. Trajectories are plotted for the longest section of flight for which the relative error remains below the threshold value of $\varepsilon \leq 0.012$, displaying the subset of 15 flights with the longest simulations meeting this criterion. Grid spacing: 10 m.

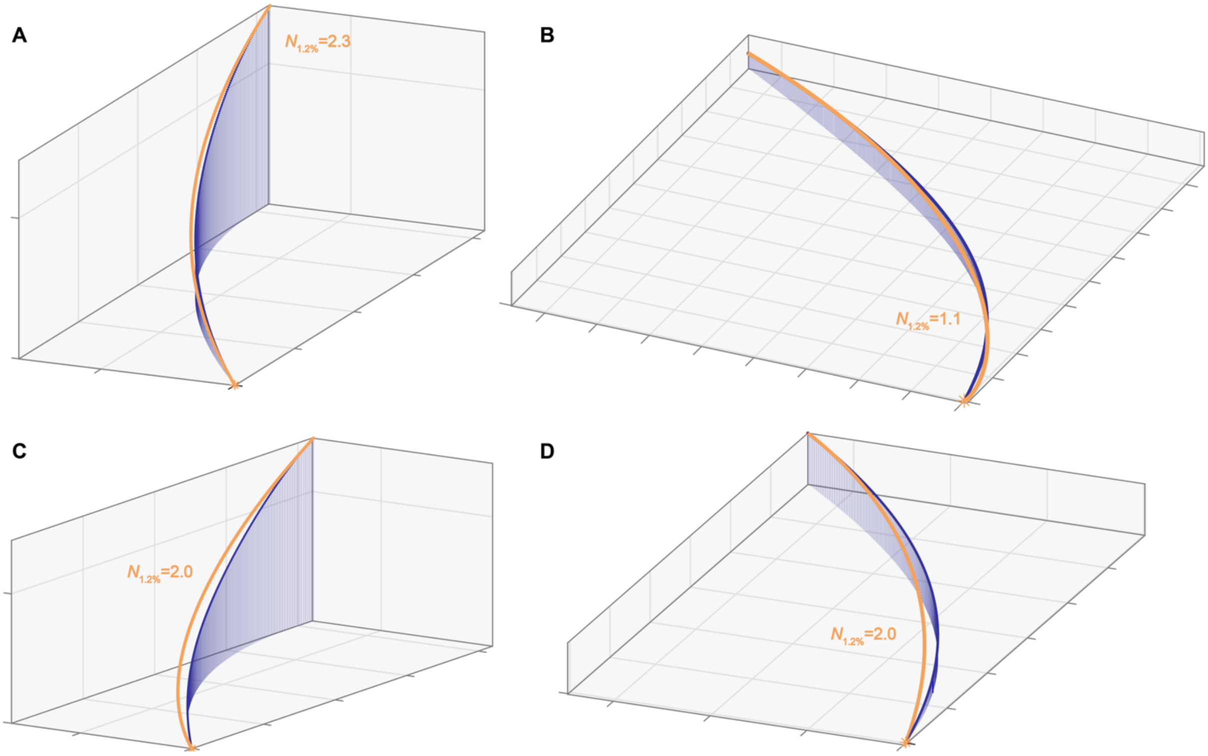


**Figure S2. Long-range approaches of Peregrine or Prairie Falcons attacking Mexican Free-tailed Bats modelled under delay-free PN targeting a fixed point in the swarm.** Each panel plots the reconstructed three-dimensional attack trajectory of an incoming falcon (dark blue points), where panels (A,C) and (B,D) represent the same trajectory fitted under a different guidance model; orange starburst shows the point of capture or near-miss. Dark blue lines are dropped vertically from each point to accurately convey the three-dimensional shape of the trajectory. It was not possible to track the bats that the falcons grabbed at this range, but orange lines plot simulations of the falcon's flight trajectory generated under delay-free PN guidance assuming flight at the same speed as measured and treating the falcon's final position as the target at: **(A,B)** the best-fitting value of the navigation constant $N$; **(C,D)** a fixed navigation constant of $N = 2$. The latter serves to generate a constant radius turn that satisfies the kinematic constraint of passing through the falcon's initial and final positions whilst also matching its initial flight velocity. Trajectories are plotted for the longest section of each flight for which the relative error remains below the threshold value of $\varepsilon \leq 0.012$. Grid spacing: 10 m.

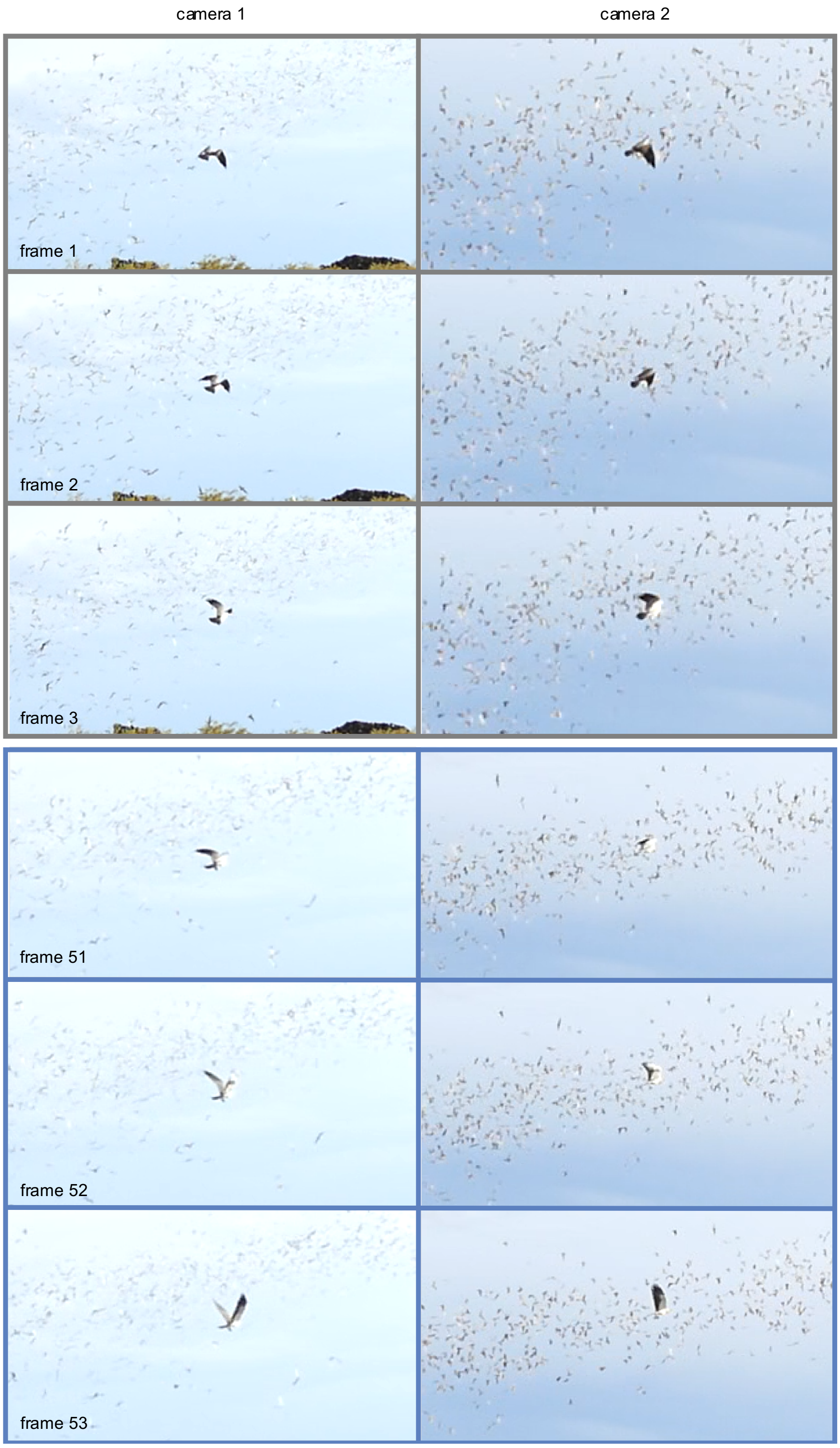


**Figure S3. Example of frame synchronisation between cameras.** The upper three rows show the frames used to synchronise the two video cameras, which were aligned to the nearest frame by reference to the observed wingbeat kinematics. The lower three rows show the kinematics recorded 50 frames (i.e. 1 s) later, confirming that the frame synchronization remains stable over this interval. Note that this procedure only guarantees synchronization of the frames to within ± 0.01 s at the 50 Hz frame rate, which is reflected in the fact that the wing pose appears similar, but not identical, between the two videos. All frames shown are from the original video collected by the authors in this study.

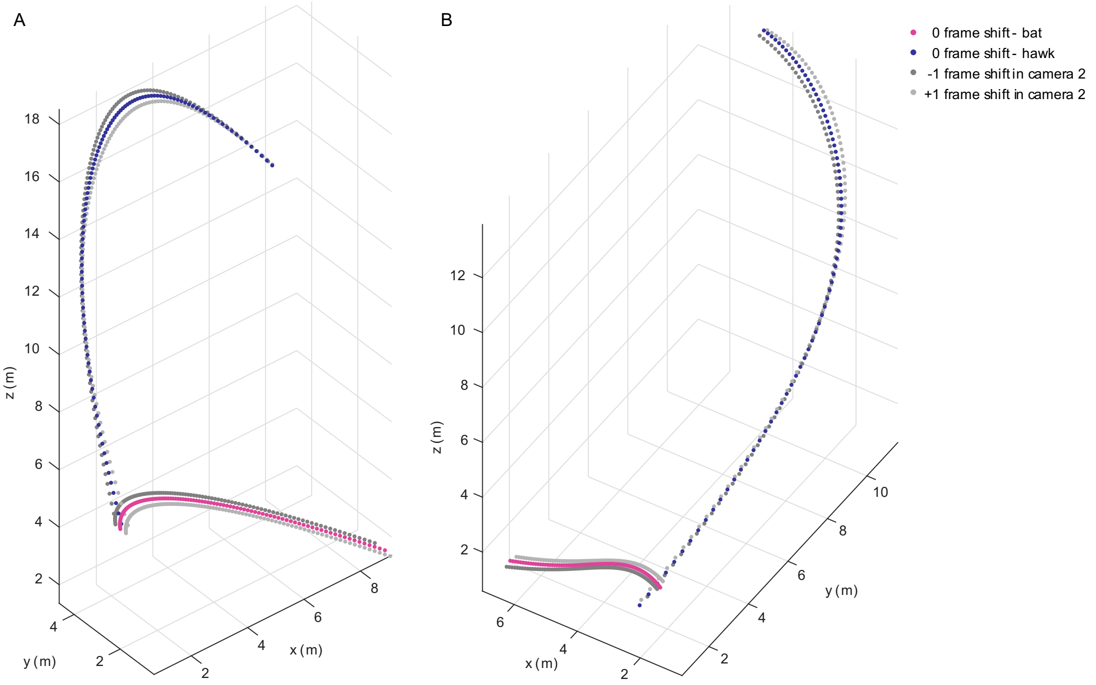


**Figure S4. Sensitivity analysis assessing the effect of camera synchronization error on the trajectory reconstructions.** Reconstructed flight trajectories of the hawk (blue) and the bat it attacks (magenta) are shown for two example attacks (A,B), together with the reconstructed flight trajectories that result from applying a shift of ±1 frame to the synchronization of the two cameras (light or dark grey). The resulting displacement of the trajectories is small in comparison to their path length, and their shape remains approximately the same. Since the cameras are reliably synchronized to the nearest frame (i.e. to within ± 0.01 s), the displacement shown here for a shift of ±1 frame (i.e. for an applied ± 0.02 s shift) will overstate the error associated with the imperfect synchronization of the cameras by about a factor of two.

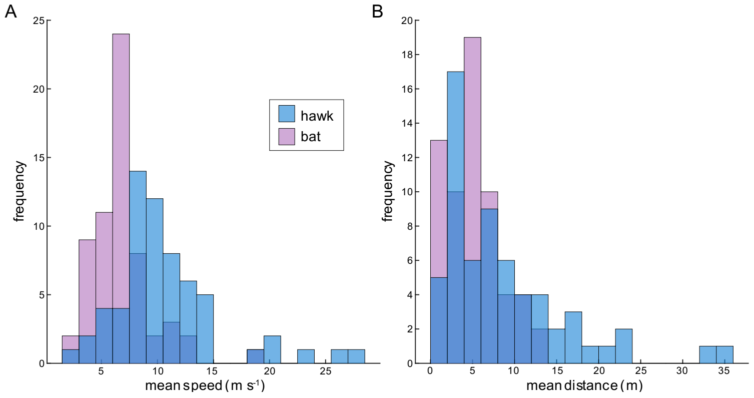


**Figure S5. Histograms summarizing measured flight performance of hawks and bats by flight. (A)** Mean flight speed. **(B)** Mean path length. Histograms display data for all $n = 62$ terminal attack trajectories.

**Table S1. Summary of fitted guidance models.** Performance of delay-free guidance models fitted to the $n = 62$ terminal attack trajectories of Swainson's Hawks attacking swarming Mexican Free-tailed Bats, for the different combinations of guidance law (i.e. PN, PP or PN+PP) and target definition (i.e. instantaneous bat position, final bat position, final hawk position). Tilde notation denotes the median values of the guidance constants $N$ or $K$, and relative error $\varepsilon$, together with the corresponding bias-corrected and accelerated bootstrap 95% confidence interval (CI).

| **target of guidance** | **fitted guidance law** | | | | | | |
|---|---|---|---|---|---|---|---|
| | **PN** | | **PP** | | **PN+PP** | | |
| | $\tilde{N}$ **(95% CI)** | $\tilde{\varepsilon}$ **(%) (95% CI)** | $\tilde{K}$ **(s$^{-1}$) (95% CI)** | $\tilde{\varepsilon}$ **(%) (95% CI)** | $\tilde{N}$ **(95% CI)** | $\tilde{K}$ **(s$^{-1}$) (95% CI)** | $\tilde{\varepsilon}$ **(%) (95% CI)** |
| **instantaneous bat position** | 1.63 (1.08, 2.04) | 2.08 (1.67, 2.68) | 0.66 (0.13, 1.62) | 2.97 (2.34, 4.15) | 1.83 (1.17, 2.57) | 0.36 (-0.29, 0.66) | 0.88 (0.64, 1.09) |
| **final bat position** | 1.65 (1.45, 2.07) | 1.09 (0.89, 1.34) | 3.06 (2.71, 4.03) | 1.23 (1.03, 1.57) | 1.96 (1.28, 3.16) | 0.24 (-1.10, 0.54) | 1.04 (0.80, 1.22) |
| **final hawk position** | 1.91 (1.81, 2.01) | 0.04 (0.03, 0.08) | 4.36 (3.51, 5.13) | 0.30 (0.24, 0.43) | 2.08 (2.03, 2.16) | -0.77 (-1.17, -0.41) | 0.01 (0.00, 0.01) |

**Table S2. Validation of the principle of using model selection to identify the target of PN guidance.** Re-analysis of published data for Peregrines and Gyrfalcons pursuing singleton aerial targets [1, 2], showing the results of fitting delay-free PN guidance targeting either the instantaneous or final position of the target. To quantify how well each model fits the data, we identify the path length and duration of the longest section of each flight for which the relative error remains below the threshold value of $\varepsilon \leq 0.012$, up to and including the point of intercept. As expected, the data are better modelled by taking account of the full information on target position, in contrast to our results for Swainson's Hawks attacking swarming bats.

| **target of PN guidance** | **median path length modelled (m) (Q1, Q3)** | | **median duration modelled (s) (Q1, Q3)** | |
|---|---|---|---|---|
| | **Peregrines** | **Gyrfalcons** | **Peregrines** | **Gyrfalcons** |
| **instantaneous target position** | 105.8 (44.8, 194.3) | 35.1 (21.6, 89.6) | 5.4 (3.1, 11.6) | 4.6 (3.2, 8.2) |
| **final target position** | 86.4 (53.4, 104.9) | 27.1 (21.0, 36.1) | 4.7 (4.1, 5.5) | 3.6 (2.8, 5.2) |

**Table S3. Contingency table summarizing success of attacks by Swainson's Hawks on swarming Mexican Free-tailed Bats.** Bats classified as flying outside the swarm column were judged to be flying > 5 body lengths from their nearest neighbour and/or appeared to be flying in a different direction to its coordinated members. Bats not meeting these criteria were classified as flying within the swarm column. Attacks are classified as successful if the hawk succeeded in grabbing the bat in its talons.

| | successful captures | failed captures | *totals* |
|---|---|---|---|
| attacks on bats flying outside swarm column | **3** | **5** | *8* |
| attacks on bats flying within swarm column | **12** | **42** | *54* |
| *totals* | *15* | *47* | *62* |

**References**


1. Brighton, C.H., et al., *Attack behaviour in naive gyrfalcons is modelled by the same guidance law as in peregrine falcons, but at a lower guidance gain.* Journal of Experimental Biology, 2021. **224**(5).
2. Brighton, C.H., A.L.R. Thomas, and G.K. Taylor, *Terminal attack trajectories of peregrine falcons are described by the proportional navigation guidance law of missiles.* Proceedings of the National Academy of Sciences of the United States of America, 2017. **114**(51): p. 13495-13500.
