# Supplementary material for: Raptors avoid the confusion effect by targeting fixed points in dense aerial prey aggregations
Source: Nat Commun. 2022 Aug 23;13:4778. doi: 10.1038/s41467-022-32354-5 (PMC9399121; doi:10.1038/s41467-022-32354-5)
Supplement: Supplementary file 3 — Description of Additional Supplementary Files [file 41467_2022_32354_MOESM3_ESM.pdf]

Description of Additional Supplementary Files

File Name: Supplementary Movie 1
Description: Sample video footage of two Swainson's Hawks Buteo swainsoni attacking swarming Mexican Free-tailed Bats Tadarida brasiliensis. Video recorded in full HD (1920×1080 pixels) at 50 Hz frame rate. The three-dimensional trajectories of the hawks and bats were reconstructed by tracking them across stereo video pairs (see Methods).
