# Supplementary material for: Raptors avoid the confusion effect by targeting fixed points in dense aerial prey aggregations
Source: Nat Commun. 2022 Aug 23;13:4778. doi: 10.1038/s41467-022-32354-5 (PMC9399121; doi:10.1038/s41467-022-32354-5)
Supplement: Supplementary file 5 — Reporting Summary [file 41467_2022_32354_MOESM5_ESM.pdf]

nature portfolio

Corresponding author(s): Graham K. Taylor

Last updated by author(s): Jul 8, 2022

Reporting Summary

Nature Portfolio wishes to improve the reproducibility of the work that we publish. This form provides structure for consistency and transparency in reporting. For further information on Nature Portfolio policies, see our Editorial Policies and the Editorial Policy Checklist.

Statistics

For all statistical analyses, confirm that the following items are present in the figure legend, table legend, main text, or Methods section.

n/a	Confirmed	
☐	☒	The exact sample size (*n*) for each experimental group/condition, given as a discrete number and unit of measurement
☐	☒	A statement on whether measurements were taken from distinct samples or whether the same sample was measured repeatedly
☐	☒	The statistical test(s) used AND whether they are one- or two-sided<br>*Only common tests should be described solely by name; describe more complex techniques in the Methods section.*
☒	☐	A description of all covariates tested
☐	☒	A description of any assumptions or corrections, such as tests of normality and adjustment for multiple comparisons
☐	☒	A full description of the statistical parameters including central tendency (e.g. means) or other basic estimates (e.g. regression coefficient) AND variation (e.g. standard deviation) or associated estimates of uncertainty (e.g. confidence intervals)
☐	☒	For null hypothesis testing, the test statistic (e.g. *F*, *t*, *r*) with confidence intervals, effect sizes, degrees of freedom and *P* value noted<br>*Give P values as exact values whenever suitable.*
☒	☐	For Bayesian analysis, information on the choice of priors and Markov chain Monte Carlo settings
☒	☐	For hierarchical and complex designs, identification of the appropriate level for tests and full reporting of outcomes
☐	☒	Estimates of effect sizes (e.g. Cohen's *d*, Pearson's *r*), indicating how they were calculated

Our web collection on statistics for biologists contains articles on many of the points above.

Software and code

Policy information about availability of computer code

Data collection	No software was used in collecting the video data described in this study.
Data analysis	All data analysis was done in MATLAB R2020a (MathWorks Inc., Natick, MA). Single camera calibration was done automatically using the Single Camera Calibrator app in MATLAB R2020a. Manual digitization of image points was done using the DLTdv5 digitizing tool in MATLAB (https://doi.org/10.1088/1748-3182/3/3/034001). Stereo camera reconstruction was done using a nonlinear bundle adjustment implemented in custom code (https://doi.org/10.1098/rsif.2008.0245) using the MATLAB Optimization Toolbox R2020a. Flight trajectory simulations were generated using custom code in MATLAB R2020a (https://doi.org/10.1038/s41467-019-10454-z). All custom code has been uploaded to figshare https://doi.org/10.6084/m9.figshare.19196966.

For manuscripts utilizing custom algorithms or software that are central to the research but not yet described in published literature, software must be made available to editors and reviewers. We strongly encourage code deposition in a community repository (e.g. GitHub). See the Nature Portfolio guidelines for submitting code & software for further information.

Data

Policy information about availability of data

All manuscripts must include a data availability statement. This statement should provide the following information, where applicable:

- Accession codes, unique identifiers, or web links for publicly available datasets
- A description of any restrictions on data availability
- For clinical datasets or third party data, please ensure that the statement adheres to our policy

Calibration images, digitized image coordinates, and trajectory reconstructions are available as Supporting Data at https://doi.org/10.6084/m9.figshare.19196966. Source Data are provided with this paper for Figures 2 and 6. Raw video data are stored locally on account of their size (0.2 TB) and will be made available upon

reasonable request.

Field-specific reporting

Please select the one below that is the best fit for your research. If you are not sure, read the appropriate sections before making your selection.

☐ Life sciences ☐ Behavioural & social sciences ☒ Ecological, evolutionary & environmental sciences

For a reference copy of the document with all sections, see nature.com/documents/nr-reporting-summary-flat.pdf

Ecological, evolutionary & environmental sciences study design

All studies must disclose on these points even when the disclosure is negative.

Study description	We filmed attacks by wild Swainson's Hawks (Buteo swainsoni) on swarming Mexican Free-tailed Bats (Tadarida brasiliensis), using three pairs of high-definition video cameras fixed in stereo configuration around the cave from which the bats emerged. We tracked the hawks and the bats that they attacked manually in the video data, and used this to reconstruct their three-dimensional flight trajectories using stereo camera reconstruction techniques. We then fitted numerical simulations to the measured flight trajectories under different hypothesised guidance laws commanding the model hawk's steering behaviour.
Research sample	The research sample was drawn from a population approximately 20 Swainson's Hawks (Buteo swainsoni) hunting Mexican Free-tailed Bats (Tadarida brasiliensis) in a maternal colony comprising approximately 700,000 to 900,000 individuals inhabiting the Jornada Caves, Armendaris Ranch, New Mexico, USA.
Sampling strategy	This was an observational study the sampling strategy of which was to record as many attacks as possible using three fixed pairs of high-definition video cameras mounted on tripods around the caves (called North and South) from which the bats emerged. We set up two camera pairs facing approximately north and south across the South cave for the duration of the study. As the swarm's overall flight direction was variable and influenced by the wind, we positioned the north- and south-facing camera pairs to allow them to be panned from northeast to northwest and from southeast to southwest, respectively. This enabled us to cover most flight directions, except due east (where the bats rarely flew) and due west (which was subject to glare). We set up a third camera pair to view the emergence that occurred from the North cave from the second week onward. When leaving the North cave, the bats usually flew along the lava tube and beneath a rock arch before climbing out of the canyon. We therefore positioned the cameras close to where the swarm began climbing out above the canyon rim, aiming to capture attacks as the hawks swooped low over the canyon.
Data collection	The bats emerged at a variable time between approximately 18:30 and 20:00 MDT. Each emergence lasted from 10 to 25 minutes, depending on the number of bats present, which increased over the course of the study. As soon as the bats began emerging, the cameras were turned on and left to record, having oriented them in the direction of the swarm's emergence, which varied from day to day. All authors were involved in the collection of the data.
Timing and spatial scale	We recorded video of the hawks attacking the bats every evening from 8 to 29 June 2018, except for one evening that had to be missed due to bad weather. The data are collected within the immediate vicinity of the North and South caves from which the bats emerged.
Data exclusions	No video data were excluded from the analysis. Attacks were identified by watching the videos after collection, and we aimed to analyse all of the attacks in which the attacking hawk was visible in both cameras.
Reproducibility	Qualitatively the same behaviours were observed by the authors in each of the two preceding years, but these were not filmed systematically.
Randomization	As this was an observational field study, we sought to analyse the totality of all the attacks that we observed. Randomization was therefore relevant only to the construction of the bootstrap confidence intervals presented in relation to the guidance parameters fitted in the trajectory simulations.
Blinding	Blinding was not possible owing to the need for the video data to be watched and digitized by the authors of the study. However, the basis of this study is to reconstruct the three-dimensional attack trajectories of the hawks, which are then compared to the results of numerical simulations under different candidate guidance laws. It follows that there is little risk of researcher bias, except in the identification of attacks for analysis, as the statistical analysis is far removed from the digitization of the video data.

Did the study involve field work? ☒ Yes ☐ No

Field work, collection and transport

Field conditions	Data were recorded under dry conditions within the 2h before sunset.
Location	Jornada Caves, Sierra County, New Mexico, USA
Access & import/export	Access to the Jornada Caves was provided by Armendaris Ranch Inc. No samples were collected in the course of this study.
Disturbance	To begin with, all fieldworkers retreated into make-shift hides as the bats began emerging, but these were gradually phased out for reasons of practicality. The birds quickly became habituated to the fieldworkers' presence, venturing close to the cave even when fieldworkers were present.

Reporting for specific materials, systems and methods

We require information from authors about some types of materials, experimental systems and methods used in many studies. Here, indicate whether each material, system or method listed is relevant to your study. If you are not sure if a list item applies to your research, read the appropriate section before selecting a response.

Materials & experimental systems

n/a	Involved in the study
☒	☐ Antibodies
☒	☐ Eukaryotic cell lines
☒	☐ Palaeontology and archaeology
☐	☒ Animals and other organisms
☒	☐ Human research participants
☒	☐ Clinical data
☒	☐ Dual use research of concern

Methods

n/a	Involved in the study
☒	☐ ChIP-seq
☒	☐ Flow cytometry
☒	☐ MRI-based neuroimaging

Animals and other organisms

Policy information about studies involving animals; ARRIVE guidelines recommended for reporting animal research

Laboratory animals	N/A
Wild animals	This was an observational field study of wild Swainson's Hawks (Buteo swainsoni) and wild Mexican Free-tailed Bats (Tadarida brasiliensis). No animals were caught in this study.
Field-collected samples	N/A
Ethics oversight	This work was reviewed and approved by the Animal Welfare and Ethical Review Board of the Department of Zoology, University of Oxford. No ethical issues were identified. As an observational study, this work was not subject to approval by the Institutional Animal Care and Use Committee at Saint Mary's College.

Note that full information on the approval of the study protocol must also be provided in the manuscript.
